# Spatiality in Health: The Distribution of Health Conditions Associated with Electronic Waste Processing Activities at Agbogbloshie, Accra

**DOI:** 10.5334/aogh.2630

**Published:** 2020-03-18

**Authors:** Abenaa Adusei, John Arko-Mensah, Mawuli Dzodzomenyo, Judith Stephens, Afua Amoabeng, Saskia Waldschmidt, Katja Löhndorf, Kwame Agbeko, Sylvia Takyi, Lawrencia Kwarteng, Augustine Acquah, Paul Botwe, Prudence Tettey, Andrea Kaifie, Michael Felten, Thomas Kraus, Thomas Küpper, Julius Fobil

**Affiliations:** 1 University of Ghana School of Public Health, GH; 2 RWTH Aachen Technical University, DE

## Abstract

**Background:**

A walk through the Agbogbloshie e-waste recycling site shows a marked heterogeneity in the spatial distribution of the different e-waste processing activities, which are likely to drive clustering of health conditions associated with the different activity type in each space.

**Objective of study:**

To conduct a spatial assessment and analysis of health conditions associated with different e-waste activities at different activity spaces at Agbogbloshie.

**Methods:**

A choropleth showing the various activity spaces at the Agbogbloshie e-waste site was produced by mapping boundaries of these spaces using Etrex GPS device and individuals working in each activity spaces were recruited and studied. Upon obtaining consent and agreeing to participate in the study, each subject was physically examined and assessed various health outcomes of interest via direct physical examination while characterizing and enumerating the scars, lacerations, abrasions, skin condition and cuts after which both systolic and diastolic blood pressure values were recorded alongside the administration of open and close ended questionnaires. All individuals working within each activity space and consented to participate were recruited; giving a total of one hundred and twelve (112) subjects in all.

**Results:**

A study of the choropleth showed that health conditions associated e-waste processing activities were clustered in a fashion similar to the corresponding distribution of each activity. While a total of 96.2% of all the study subjects had cuts, the dismantlers had higher mix of scars, lacerations and abrasions. Abrasions were observed in 16.3% of the dismantlers. Scars were the most common skin condition and were observed on the skins of 93.6% of the subjects. Prevalence of burns among the study subjects was 23.1%. Developing hypertension was not associated with activity type and while a total of 90.2% of subjects had normal blood pressure and 9.8% of them were hypertensives. Finally, 98.2% of respondents felt the need to have a first aid clinic at the site with 96.4% and 97.3% willing to visit the clinic and pay for services respectively.

**Conclusion:**

We conclude that while the observed injuries were random and were due purely to accidents without any role of spatial determinants such as the configuration, slope, topography and other subterranean features of the activity spaces, a strong association between the injuries and activity type was observed.

## 1. Background

The United Nations Environment Program, UNEP (2005) estimates that between 20 and 50 million tonnes of e-waste are generated annually worldwide, accounting for about 5% of all municipal solid waste. In a recent global waste stream analysis, the composition of global quantity of e-waste generated in 2014 comprised of 1.0 Mt of lamps, 3.0 Mt of Small IT, 6.3 Mt of screens and monitors, 7.0 Mt of temperature exchange equipment (cooling and freezing equipment), 11.8 Mt of large equipment, and 12.8 Mt of small equipment and the global is projected to grow to 49.8 Mt in 2018, with an annual growth rate of 4 to 5 per cent [1,2]. Not only is this figure representing the fastest growing municipal waste stream, it also has the potential of increasing further. In spite of the unprecedented growth in the global quantities, there is only limited recycling technology for disposal and safe management especially in the developing countries where most of the wastes end up and are recycled by informal means using rudimentary methods [3,4].

In Ghana for example, before the arrival of electronic waste at Agbogbloshie, the area was a wetland known as Old Fadama and predominantly a place for selling agricultural food products. It was the place of escape for refugees running from the Kokomba and Nanumba (located in the Northern part of Ghana) conflict [3]. The Agbogbloshie area which is now a major site for informal e-waste recycling is less than one kilometre from the Central Business District of Accra and is about thirty-one hectares in size [3,5,6]. It is bounded south, west and Northwest by the Odaw River, which feeds into the Korle Lagoon. There is a popular yam and onion wholesale market close to the e-waste recycling site. In totality, the scrap yard takes up about one hectare [7]. The recycling site has emerged as an interesting case study for many reasons including that it is a hub of global sink for used electronic products where recovery of products from the waste stream has multiple adverse impacts on the environment and human health. Ongoing research work at the site has shown that recycling and product recovery activities emit huge quantities of toxic chemical mixtures into the ambient environment [4,8,9]. Known widely for a mix of both formal and informal economic activities, which co-exist in an environment charged with tensions among the economic actors, ongoing research has established that toxic chemical mixtures often generated during e-waste recovery activities resulting in high levels of exposures which are affecting a broad spectrum of the urban population, especially among the e-waste recovery workers [4,6,10,11].

At the recycling location, there are various activities specific to each part of the site, though one would also observe that certain activities cut across the entire recycling area. For example, there are specific places one would see only dismantling of fridges, air conditioners, car parts, televisions and computers taking place but sorting of wires seems to run across the entire site. The e-waste recycling site therefore shows a striking heterogeneity of spatial patterns of the different e-waste processing activities, which are likely to drive clustering of physical injuries and other health conditions associated with the different activity type in space. Skin conditions were classified into scars, rashes, peeling and burns. In this study, a Global Positioning System (GPS) device (Etrex 10 GPS: Garmin, Kansas City) was used to demarcate the area into activity spaces specific to recycling of specific recovered products from the waste stream. The aim was to study the recyclers working in those activity spaces in order to estimate the burden of various physical injuries and health outcomes such as scars, rashes, skin peeling, and burns unique to the recycling activities in each demarcated area and across space. The study estimated the prevalence of injuries using markers such as cuts, abrasions, avulsions and lacerations sustained by the workers during e-waste processing/recycling as they performed these activities. Finally, the study also assessed blood pressure levels among the e-waste workers in each demarcated activity space in order to determine if blood pressure levels differed across each worker class and activity space because of pervasively stressful nature of the recycling environment.

## 2. Materials and methods

### 2.1. Study Area

The study was conducted at the Agbogbloshie e-waste site which is arguably one of the largest e-waste dumps in the world. Agbogbloshie, known also as Old Fadama, serves as a home to some 40,000 people who are among the world poorest urban populations [3,4]. It is one of the biggest slums ever created by urbanization in West Africa [3,4,5,12]. It is bounded eastward by the Korle River and westward by the highly polluted Odaw River which feeds into the Korle Lagoon on the south-side. Agbogbloshie serves not just a home to thousands of informal sector workers and site for e-waste recycling, it is also noted for the popular Agbogbloshie Market where all major food products and farm produce are sold; popular farm produce are onions, tomatoes, vegetables and yams [3]. This draws thousands of residents of the city to the area on a daily basis – a principal concern to health authorities because, the widespread environmental pollutants due to informal level e-waste recycling activities are likely to impact adversely on a wider population and pupils of the near-by basic schools [3,5].

### 2.2. Participant Recruitment

Various activity spaces were mapped using an Etrex geographic positioning system (GPS). The workers were found mainly in small groups at the various activity spaces. Since there were not many e-waste workers per activity space at the site, all workers who were working and willing to participate in the study were recruited and included in the study. In total, 112 individuals located in the different activity spaces who were involved with the collection, sorting, dismantling and burning of e-waste materials were therefore included in the study.

### 2.3. Study Procedures

In this study, we demarcated the entire recycling area into subunit spaces in which similar recycling activities took place and defined by homogenous activity patterns using a Global Positioning System (GPS) device Etrex 10 GPS (Garmin, Kansas City); making use of longitudes and latitudes. We then recruited the recovery workers performing recycling activities in the demarcated areas after they had consented to participate in the study and then conducted direct examination of the skin for injuries and other visible skin conditions as markers of previous injuries to the skin. In a simple survey, we asked questions about their age, level of education, marital status, number of months/years in this recycling job, and number of hours on the job per day. We also measured and recorded systolic and diastolic blood pressure of all study subjects in order to estimate the prevalence of stress related cardiovascular conditions among the recyclers as it is widely reported that the e-waste workers conduct their activities under very stressful environment [13]. For this reason, a questionnaire was employed to collect demographic data on all participants and to elicit responses from the subjects as well as collect information on their knowledge and use of personal protective equipment (PPE). This helped in assessing injuries profile on the skin as well as whether or not a first aid clinic was a need in the area. A translator and three other trained research assistants assisted with questionnaire administration. All study participants were assigned a unique identity (ID) comprising a prefix and a number. The prefixes were defined by the activities performed by the subjects. The following prefixes were used:

- COR – Collecting
- SOR – Segregation
- DIS – Dismantling
- BUR – Burning.

Factors such as age, history of high blood pressure, old scars before starting work, injuries and burns not sustained at the e-waste site were assessed to control for possible confounding.

Physical examination of the skin was conducted to numerate scars over skin as an indicator of injuries and these were classified into the different types of injuries which were studied. Scars are broken patches in the skin indicating points of skin break due to cuts, puncture, sore, burns and other forms of injuries to the skin. During physical examination, we counted the on hands, legs and over the entire body-surface. Workers identified and differentiated between scars which were sustained during e-waste work and those not associated with e-waste work. Systolic and diastolic blood pressure of all study participants were checked three consecutive times with a calibrated digital Omron Blood Pressure Monitor, by Omron Corporation, Japan. The mean was found for both systolic and diastolic blood pressure for each study subjects in accordance with World Health Organization (WHO) standard for diagnosing hypertension.

### 2.4. Data Analysis

Points taken with the GPS device were entered into excel to generate longitudes and latitudes and these were exported into ArcGIS 10.1 for mapping. The completed questionnaires were crosschecked by the study team. These were all entered into Epi Info^TM^ 7 software. They were then entered into Microsoft Excel spread sheet which were subsequently exported to Stata Version 12. The data were cleaned and validated before analyses were conducted.

### 2.5. Statistical Procedures

The use of Pearson’s Chi square was employed to test for differences in proportions across the different groups defined by the distinct activity spaces. Fischer’s exact p-value was determined where cell count was low; below 5. Analysis of variance (ANOVA) was conducted to determine if differences existed across groups for continuous variables.

## 3. Results

Findings of this study are presented to highlight the relationship between the different e-waste processing activities that take place at the Agbogbloshie dumpsite and physical injuries as well as other skin conditions that the e-waste workers experience.

### 3.1. Mapping of different e-waste processing activities

Table 1 shows the geocodes and coordinates of the regions within which specific e-waste processing activities took place in space at recycling site.

- WB – West Bank, situated on the boundary line of Abossey Okai road where it intersects with the Odaw River – a region where the predominant activity is sorting.
- FA – Fridge area – Marks the beginning of the burning area.
- SE – South end – representing heavy burning area.
- SCP – South Central Point, represents a region of minimal burning.
- SCWB – South Central West Point – represents region of predominant dismantling.
- SWE – South West End – region close to Central Gospel Church where sorting and some dismantling took place.
- MWE – Middle West End – region close to the football park where predominantly dismantling and minimal sorting took place.
- BIF – a region near the Blacksmith Institute Facility where true recycling/refurbishing of recovered products took place.
- NEW – North End West – a region close to the boundary line of the Abossey Okai road where sale of foodstuff is predominant activity.
- NCP – North Central Point – a region where main activity is dismantling.
- WBE – West Bank End.

**Table 1:** Coordinates of mapped out areas.

Description	X (Longitude)	Y (Latitude)

BIF	–0.22478	5.553944
FA	–0.22653	5.552306
MWE	–0.22447	5.553111
NCP	–0.225	5.553778
NEW	–0.22642	5.554167
SCP	–0.22669	5.551056
SCWB	–0.22522	5.551528
SE	–0.22731	5.550583
SWE	–0.224	5.552472
WB	–0.22397	5.553278
WBE	–0.21667	5.553278

**Figure 1:**
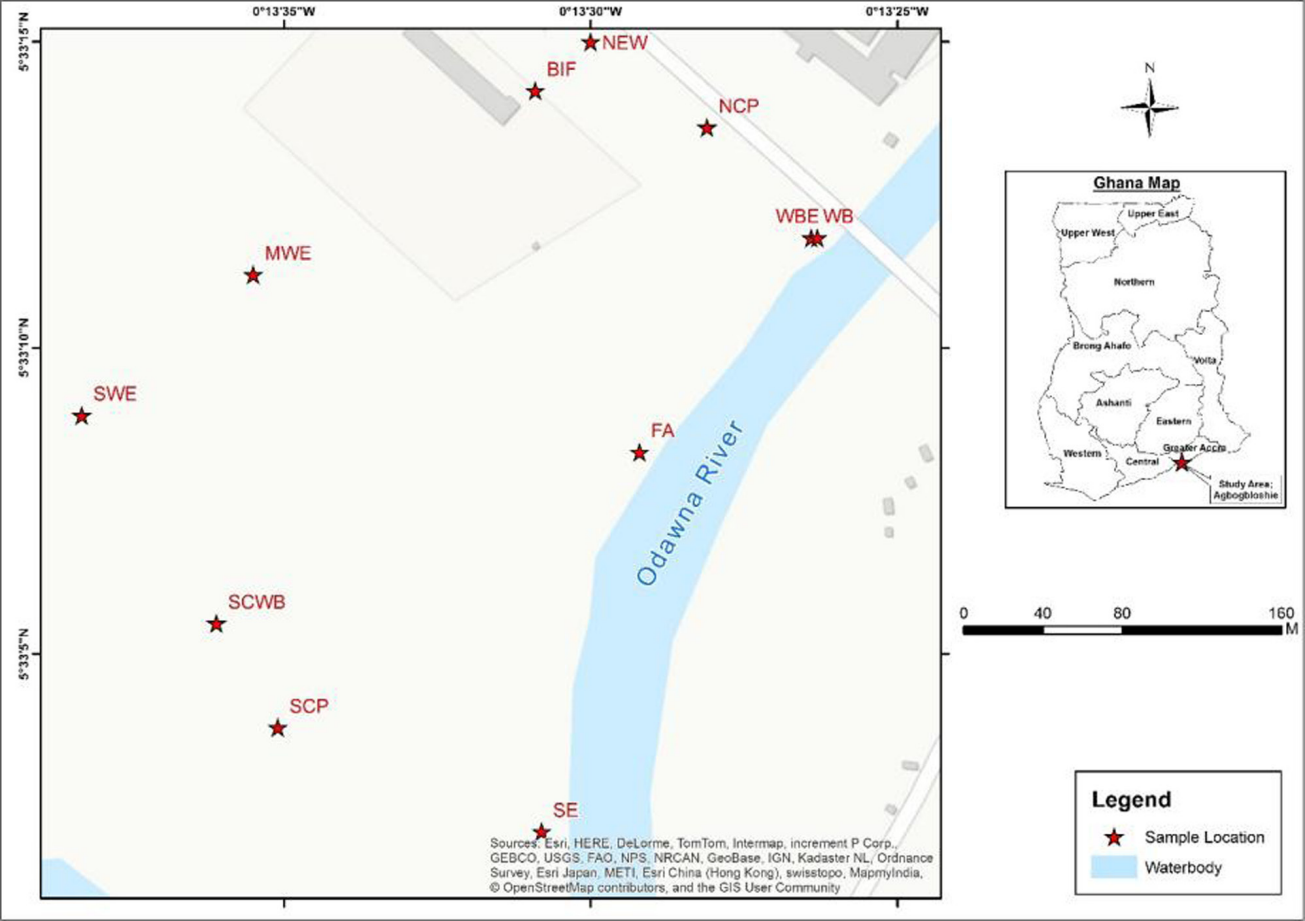
Spatial distribution of e-waste processing activities at Agbogbloshie.

Figure 1 shows choropleth or map of activity areas produced using the geo-coordinates presented in Table 1 below.

As seen on the choropleth, regions WB, FA, SE and WBE were mapped along the banks of Odaw River while regions NEW, WB, WBE and NCP bounded the Abossey Okai road.

This was predominantly an area for sorting and the observed health conditions were mostly cuts, lacerations, scars and rashes. The area marked as FA (fridge area) marked the beginning of e-waste burning, which increased in intensity toward the area marked as SE (South End) where heavy burning activities took place. The observed injuries were cuts, lacerations, abrasions, burns, scars, rashes and skin peeling. Within the region described as the SCP (south central point), some minimal burning was observed and the corresponding predominant health conditions observed were burns, scars and cuts. In the area marked as SCWP (south central west point), dismantling was the predominant activity and the commonest the observed physical injuries were mainly cuts, lacerations, scars rashes and burns. Sorting and some dismantling were major processing activities observed at the are labelled SWE (South West End) and the corresponding reported physical injuries were cuts, lacerations, scars and rashes. In the region marked as MWE (Middle West End), predominantly dismantling and some sorting took place. Again, the physical reported were mostly cuts, laceration, scars and rashes. The region marked as BIF (Blacksmith institute’s facility) where the association office is located, fabrication of e-waste materials into cooking wares and pots (popularly called “gyapa” in Ghana) was observed, but no subjects were recruited from this area. The area marked as NWE (North End West) which bordered the Abossey Okai road and the area marked as NCP (north central point) housed sales of farm produce and refurbishing activities. The most dominant health conditions observed in this area were cuts, abrasions and scars, although less pervasive compared to other activity spaces.

### 3.2. Demographic Characteristics of the Study Participants

One hundred and twelve (112) e-waste workers at different activity spaces comprising Collectors, Sorters, Dismantlers and Burners were recruited into the study. The demographic profile of the participants is shown in Table 2 below.

**Table 2:** Demographic characteristics of study subjects.

Characteristic	Activity space					P-value

	Collecting	Sorting	Dismantling	Burning	Total	

**Age**						*P* = *0.010*
**<20**	3 (8.6)	1 (6.3)	7 (18.0)	9 (40.9)	20 (17.9)	
**20–29**	22 (62.9)	7 (43.8)	23 (59.0)	11 (50.0)	63 (56.3)	
**30 – 39**	8 (22.9)	6 (37.5)	8 (20.5)	0 (0.0)	22 (19.6)	
**40+**	2 (5.7)	2 (12.5)	1 (2.6)	2 (9.1)	7 (6.3)	
**Mean (SD)**	26.1 (6.8)	28.1 (7.6)	24.5 (6.1)	22.1 (9.9)	25.3 (7.5)	
**Marital status**						
**Unmarried**	16 (45.7)	6 (37.5)	18 (46.2)	10 (45.5)	50 (44.6)	
**Married**	19 (54.3)	10 (62.5)	21 (53.9)	12 (54.6)	62 (55.4)	
**Highest education**						*P* = *0.314*
**None**	16 (45.7)	8 (50.0)	11 (28.2)	4 (18.2)	39 (34.8)	
**Primary**	9 (25.7)	4 (25.0)	13 (33.3)	10 (45.5)	36 (32.1)	
**Junior High**	3 (8.6)	0 (0.0)	6 (15.4)	4 (18.2)	13 (11.6)	
**Secondary**	7 (20.0)	4 (25.0)	8 (20.51)	3 (13.6)	22 (19.6)	
**Region**						
**Northern**	33 (94.3)	14 (87.5)	33 (84.6)	20 (90.9)	100 (89.3)	
**Others**	2 (7.1)	2 (12.5)	6 (15.4)	2 (9.1)	12 (10.7)	
**Ethnic group**						
**Dagomba**	33 (94.3)	14 (87.5)	27 (69.2)	15 (68.2)	89 (79.5)	
**others**	2 (5.7)	2 (12.5)	11 (28.2)	7 (31.8)	22 (19.6)	
**Length of work**						*P = 0.497*
**6–12 months**	4 (11.4)	1 (6.25)	4 (10.3)	4 (18.2)	13 (11.6)	
**1–5 years**	13 (37.1)	4 (25.0)	11 (28.2)	10 (45.5)	38 (33.9)	
**>5**	17 (48.6)	11 (68.8)	24 (61.5)	8 (36.4)	60 (53.6)	
**Daily working hours**						
**5–10 hours**	9 (25.7)	3 (18.8)	9 (23.8)	5 (22.7)	26 (23.2)	
**11–15 hours**	24 (68.6)	12 (75.0)	30 (76.9)	16 (72.7)	82 (73.2)	

Fischer’s exact p-value was used due to low cell count, below 5.

Characteristic background of study participants considered were sex, ages, marital status, region, tribe, educational background, length of work and daily working hours as captured in the questionnaire. All the study participants were males. The ages of the participants ranged from 16 to 55 years. Sixty three (63) out of the 112 participants representing 56.3% were in their twenties and 6.3% of them were 40 years and above. Those between 20–29 years dominated other age groups across the activity spaces. There were more married workers than the unmarried. Fifty participants (44.6%) and 62 (55.4%) were unmarried and married respectively. Thirty-nine (39) of them; representing 34.8% had no formal education whereas 32.1% had primary education with 11.6% and 19.6% having had Secondary and Junior education respectively. Majority (89.3%) hailed from the Northern Region of Ghana with 10.7% of them hailing from the other regions of the country. Eighty-nine 89 (79.5%) of study participants were of Dagomba ethnicity. There were two foreign nationals from Nigeria and Togo and while the majority of the workers had been working for 11–15 years (73.2%), 23.3% had been in the e-waste processing business for 5–10 years. In addition, whereas 53.6% had worked for more than 5 years, 33.9% and 11.6% of the workers had worked for 1–5 years and 6–12 months respectively.

### 3.3. Injury experience among e-workers in the different activity spaces

Table 3 shows injury profile of the workers within the different activity spaces. Generally, cuts were the most frequent injuries in all the activity spaces as compared to the other injuries. A majority (96.2%) of all study subjects had one form of cuts or the other. Overall, cuts were most common (94.9%) among dismantlers compared to the other groups. In particular, the burners; in whom 90.9% cuts were observed, were observed to be highly exposed to risk of fire burns in addition to being the second group most prone to injury experience. Lacerations were the second most common injury conditions observed on 46.6% of workers across the activity spaces and whereas 74.4% of dismantlers were observed to have lacerations, 54.5% of burners were observed to have had scars revealing a history of lacerations. Although abrasions were not as common among the e-waste workers compared to cuts and lacerations, 38.5% of dismantlers and 18.9% of sorters were observed to have scars indicating past experiences of this class of injuries. The burners had the highest prevalence (77.3%) of burns followed by dismantlers with 11.4% (Table 3). The burns experienced by the dismantlers could possibly be chemical burns. As they disassembled the WEEEs, the harmful chemicals could spill and get into contact with their bodies to cause various degrees of injuries to the skin, including burns.

**Table 3:** Injury experience among e-waste worker groups.

Characteristic Assessed	Activity space					Statistic P-value

	Collectors	Sorters	Dismantlers	Burners	Total	

**Injuries^b^**						
Cuts	30 (85.7)	13 (81.3)	37 (94.9)	20 (90.9)	100 (96.2)	0.799
Lacerations	10 (26.3)	5 (31.3)	29 (74.4)	12 (54.5)	56 (46.6)	0.208
Abrasions	1 (2.8)	3 (18.9)	15 (38.5)	3 (13.6)	22 (16.3)	0.038
**Total no. of cases**	35 (100.0)	16 (100.0)	39 (100.0)	22 (100.0)	112 (100.0)	

^b^ Multiple responses allowed for various injuries and skin conditions. Fischer’s exact p-value was used due to low cell count, below 5.

### 3.4. Assessment of skin conditions of workers

Table 4 presents skin conditions of workers in each worker-category. An assessment of recyclers’ skin revealed that all dismantlers (100.0%), 90.5% of burners, 96.4% of collectors and 87.5% sorters presented with scars of varying degree of density over the skin. Skin rash was most common skin conditions (28.6%) among burners compared to 10.7% among collectors and 10.5% among dismantlers.

**Table 4:** Skin conditions among the different worker-category/activity space.

Characteristic Assessed	Activity space					Statistic P-value

	Collectors	Sorters	Dismantlers	Burners	Total	

**Skin conditions^b^**						
**Rashes**	27 (96.4)	14 (87.5)	39 (100.0)	19 (90.5)	99 (93.6)	0.275
**Scars**	3 (10.7)	3 (8.5)	4 (10.5)	6 (28.6)	16 (14.6)	0.201
**Skin peeling**	0	0	3 (7.9)	1 (4.8)	4 (3.9)	0.368
**Cumulative no. of cases**	35 (100.0)	16 (100.0)	39 (100.0)	22 (100.0)	112 (100.0)	
**Burns**						<0.001
**No**	30 (93.8)	14 (93.3)	31 (88.6)	5 (22.7)	80 (76.9)	
**Yes**	2 (6.3)	1 (6.7)	4 (11.4)	17 (77.3)	24 (23.1)	
**Cumulative burns**	32 (100.0)	15 (100.0)	35 (100.0)	22 (100.0)	104 (100.0)	
**Mean Scar #s/person**	35	16	36	22	112	
**Number Mean**	17.0	21.4	30.6	27.0	24.3	
**Stand. dev**	9.8	10.5	12.1	10.9	12.3	
**P50**	16	23	32	30	25.5	
**IQR**	15	14.5	13	10	18	

^b^ Multiple responses allowed for various injuries and skin conditions. Fischer’s exact p-value was used due to low cell count, below 5. Stand dev – standard deviation, P50 – 50^th^ Percentile, IQR-Intra Quartile Range.

Generally, skin peeling was low among all worker-groups and while the proportion of workers observed to have this skin condition was 7.9% among dismantlers and 4.8% among burners, none was observed among collectors and sorters. Sustaining an injury and being afflicted with a given skin condition were not associated with working within any activity space. As it was anticipated, the frequency of burns was higher among burners (77.3%), than sorters (11.4%), dismantlers (6.7%) and collectors (6.3%) (Table 4). With a mean scar density of 30.6, scars on skin surface were more widespread among the dismantlers than in burners (mean scar density = 27.0), than in sorters (mean scar density = 21.4) and than in collectors (mean scar density = 17).

### 3.5. Association between injury levels and workers-characteristics

Among the different injury conditions, whereas cuts were more common among the workers as indicated in Table 5 age and marital status did not show any association with the injury conditions. However, the frequency of scars increased with respect to the length of time spent performing e-waste work and number of hours/day spent working (Table 5). Overall, skin conditions did not show association with subject characteristics such as age, marital status, length of time on the job and number of hours spent per day working.

**Table 5:** Association between injury levels and worker-characteristics.

Characteristic	Injuries			Total no. of cases	P-value

	Cuts	Lacerations	Abrasions		

**Age**					0.250
<20	14 (77.8)	4 (22.2)	2 (11.1)	18	
20–29	46 (80.7)	12 (21.1)	5 (8.8)	57	
30–39	14 (82.4)	4 (23.5)	2 (11.8)	17	
40+	2 (33.3)	2 (33.3)	2 (33.3)	6	
**Marital status**					0.114
Unmarried	36 (81.8)	6 (13.6)	2 (4.6)	44	
Married	40 (74,1)	16 (29.6)	9 (16.7)	54	
**Length of work**					0.901
6–12 months	10 (83.3)	2 (16.7)	2 (16.7)	12	
1–5 years	25 (75.8)	7 (21.2)	3 (9.1)	33	
>5	40 (77.3)	13 (25.0)	6 (11.5)	52	
**Daily working/hours**					0.416
5–10 hours	15 (71.4)	5 (23.8)	1 (4.8)	21	
11–15 hours	59 (80.8)	15 (20.6)	9 (12.3)	73	

### 3.6. Assessment of Systolic and Diastolic Blood Pressure

An assessment of mean systolic and diastolic blood pressure of the workers did not show evidence of differences across the activity spaces and the workers generally had normal blood pressure levels (Table 6).

**Table 6:** Mean systolic and diastolic blood pressure across activity spaces.

Characteristic	Activity space					P-value

	Collecting	Sorting	Dismantling	Burning	Total	

**Blood pressure**						
Mean systolic Hg	123.1 ±11.3	120.7 ± 11.7	121.7 ± 12.8	119.7 ± 3.9	121.6 ± 12.3	0.773
Mean diastolic Hg	72.8 ± 8.7	73.1 ± 10.5	74.1 ± 8.7	72.4 ± 9.5	73.2 ± 9.0	0.887
**Hypertension**						0.315
Normotensive	29 (82.9)	16 (100.0)	36 (92.3)	20 (90.9)	101 (90.2)	
Hypertensive	6 (17.1)	0 (0.0)	3 (7.7)	2 (9.1)	11 (9.8)	
**Total**	35 (100.0)	16 (100.0)	39 (100.0)	22 (100.0)	112 (100.0)	

ANOVA used to test for association.

## 4. Discussion

A wide variety of hazards is associated with waste recycling industry and while quite a reasonable number is documented in the formal recycling sector, all the hazards have generally gone undocumented in the informal sector waste recycling industry. For instance, a study of consumer waste recycling in Quebec found elevated exposures to airborne bacteria, noise, carbon monoxide (during winter months only) and ergonomic hazards [14]. Other specific types of waste recycling in the formal sector have noted elevated exposures to lead in lead acid battery recycling and polybrominated diphenyl ethers in electronic recycling [15,16]. In respect of specific health outcomes, a baseline data developed over a 3-year period from 2007/08 to 2010/11 on the Occupational Health and Safety (OHS) risks associated with the e-waste recycling industry in Australia has shown that:

- 50% of injuries were cuts and lacerations primarily to hands and forearms during the disassembling process.
- 30% were sprains and strains – associated with the manual handling tasks and repetitive arm work in the disassembly process.
- 10% were bruising – mainly involved in the manual handling of the TVs and computers from the storage and disassembly processes.

Previous studies on informal sector e-waste recycling at Agbogbloshie have also reported several risk factors at play in the recycling process (Akormedi et al., 2013, Asampong et al., 2015, Yu et al., 2016), but to the best our knowledge, we report for the first time, spatial clustering of health conditions associated with the informal e-waste recycling sector.

### 4.1. Injury Types, Frequency and Distribution

Electronic waste (e-waste) recycling workers may encounter different types of hazards including the risk of injury, hearing loss, and exposure to toxic dusts and other noxious chemicals [17,18,19,20]. These hazards can cause permanent and serious health problems that could begin without workers being aware of them [14,21,22,23]. In the current study, injuries arising from e-waste processing activities which were observed among e-waste workers were classified as cuts, lacerations and abrasions. Conceivably, cuts were most common among dismantlers because of the nature of the operations associated with the dismantling process. Dismantling activity involves the use of hammer and physical force to disassemble electrical and electronic equipment component parts and for this reason; the activity is associated with frequent cuts due to the physical force applied. The most prevalent injuries were cuts, as 96.2% were observed to have scars due to cuts and it made sense because sharps were the most abundant component materials in the e-waste stream. While the level of the different injury types was observed to be a function of the type of materials handled by the e-waste workers, the frequency of the injuries could be explained by the level of safety measures in place and the level care observed by the workers. While some studies have observed that workers who engaged in recycling of cathode ray tubes (CRTs) sustained cuts than other injuries from broken tubes, others have reported that e-waste workers were at a higher risk of work related accidents and more likely to suffer physical injuries and physical disabilities than the general population [12,24,25,26,27,28,29,30]. Lacerations and abrasions were observed to be a consequence of friction-based injuries, were second (46.6%) and third (16.3%) most frequent injuries respectively and were reported to result from falls and other accidents involving falling objects pulling/sliding over the skin. The 20-29 years group had the highest proportion (80.7%) of workers with cuts, probably suggesting that they were more prone to taking higher risks rather than being less careful or while performing recycling work. This age group also had the highest number of individuals with lacerations and abrasions confirming our assertion that the observed high frequency of cuts was a consequence of increased intensity of physical activity and more tedious work rather than due to the type of materials handled. These results are not substantially different from those of a study of working conditions in a consumer waste recycling facility in Sweden in which workers reported regular exposures to noise, ergonomic hazards, falls, lifting, and awkward postures as well as regular occurrence of accidents, injuries, and pain related to ergonomic hazards [25,31,32,33].

Into-the-bargain, the length of time on the job was observed to be an important determinant of injury experience among the e-waste workers and those who had spent more than 5 years on the job sustained twice and 4 times cut injury levels compared to those who had spent 1-5 years and 6-12 months on the job respectively. Again, scar injury levels corresponded to laceration injury experience in that workers who spent more than 5 years on the job experienced twice and 4 times laceration injury levels compared to those who worked for 1–5 years and 6–12 months respectively. A plausible explanation for the observed level of injury experience is that the workers who had been on the job longer probably had become well adapted to the hazards associated with the job and were less “risk-averse” to those hazards. This assertion is supported by the observation that non-usage of personal protective equipment (PPEs) was more common in those who had spent >5 years (55.7%) as compared to 50.0% and 46.2% for those who had spent 1–5 years and 6–12 months on the job respectively. Our finding is quite consistent with that of a study which evaluated health and safety hazards at a scrap metal recycling facility in Washington State and reporting that the use of personal protective equipment and exposure controls was generally low among the recycling workers [7,15,20,23,24,25,32,34,35]. Use or non-use of PPEs has a positive relationship with worker’s perception of risk associated with the activities or tasks they undertook, which was suggestive that the use of PPEs was relatively higher among workers who had been on the job for shorter time periods. This assertion is corroborated by the observation that use of PPEs was (53.9%) among workers who had worked for 6–12 months, (50.0%) among those who had worked for 1–5 years and (45.0%) among those who had been on the job for more than 5 years [5,12,36,39]. Lastly, injuries were more common in and around the palms as compared to the feet. This meant that as a first step toward improving health and safety, hand-glove usage would be much more important than for example the wearing of safety shoes.

### 4.2. Skin Injuries, Type and Frequency

Burns did not only include burns due to fire-burns during e-waste burning process, but also chemical burns sustained as a result of coming into contact with harmful substances. The skin is the primary exposure surface to physical injuries and at the same time the natural exposure barrier to chemical agents in environmental media and therefore the skin condition serves as a marker of previous and current exposure levels. Scars being markers of physical injuries to the skin were the most common skin conditions across all worker-categories, suggesting that all categories of workers were exposed to cuts and physical injuries and dismantlers in whom scars were most common were also observed to be engaged in the most vigorous and most tedious physical activity. In unprotected workers or workers without PPEs, it would be seen that scar-density (i.e. the highest average scar-count per person) was greatest among dismantlers who used heavy tools to break apart, the components of electrical and electronic materials and therefore most prone to physical injuries which were lowest among the collectors. In terms of the frequency of skin condition, scars were commonest, followed by rashes and with skin peeling being the least common across the worker-categories. We observed no cases of skin peeling among collectors and sorters which meant that sorting and collection activities did not unduly expose the e-waste workers to harmful substances that are part of technical formulation of electrical and electronic devices and which irritate the skin. On the basis of the nature of recycling activity alone, dismantlers were more likely to come into direct contact with both elemental and inorganic mercury, nickel, beryllium, lead and several organic compounds such as flame retardants which are found in the e-waste materials and may spill due to heavy cracking of the dismantling process. Some studies have shown that Hg may cause skin rashes, skin discoloration, scarring as well as a reduction in the skin’s resistance to bacterial and fungal infections [26,29,31,36,40,41,42]. Mercury is ubiquitous in e-waste materials which is commonly found in thermostats, sensors, relays, thermometers, switches commonly found on printed circuit boards, mobile phones, batteries and in flat display panels and several studies have suggested that the use of mercury is likely to increase in flat panel displays in years to come and this will further increase the risk of exposure to mercury [7,22,43,44,45,46].

### 4.3. Spatiality, Hazard/Injury Distribution and Spatial Ordering

In spatial epidemiology, spatial frailty models are commonly used to estimate random effects – spatially explicit ordering of events such as health outcomes or any other health events occurring in space [47,48,49,50]. At Agbogbloshie, e-waste processing activities which are strongly associated with health outcomes; especially injuries that may underlie or determine the ordering of these health events across space. The observed injuries were random and were due purely to accidental events without spatial determinants such as the configuration, slope, topography, and other subterranean features. However, many of the observed health conditions tended to cluster according to the type of activities and by extension, activity spaces, e.g. underlying variations in environmental exposures and distribution of risk factors/hazards which in turn ultimately determine the distribution of the health outcomes. Scars for instance were observed to cluster within activity spaces in which the main activity in the area was dismantling, while burns tended to cluster around areas where the predominant activity was e-waste burning. On the contrary, skin condition did not show any spatial clustering suggesting that the determining factors (exposures) skin conditions were not location or space-dependent and are probably defused exposures in nature (Figure 1). This is consistent with the reports of other studies which showed that the geographical distribution of health outcomes is influenced by socio-economic and environmental factors operating at different spatial scales [21,37,51,52,53]. Spatial variability in geographic events can be revealed with semi-parametric Geographically Weighted Poisson Regression (sGWPR), models that can combine both spatially varying and spatially non-varying parameters [21,53,54,55]. Indeed, measuring spatial relationships between socio-economic and environmental factors on the one hand and health events on the other is a fairly new scientific endeavour and often very challenging; but forms a crucial part of spatial epidemiology [20,48,56]. To emphasize this point, air quality within urban environments involves a mixture of gaseous and particulate concentrations that are affected by a variety of emission sources, local topographies, and meteorological conditions [4,6,26,29,30,38,42,43,57]. As such, complex spatial patterning can occur in urban air quality making the variability of such phenomena difficult to characterize as different pollutants often exhibit differential spatial patterns (e.g., ozone vs. nitrogen dioxides) [58].

However, recent advancements made in the development of spatial methods for studying spatial variation in health outcomes have made it possible to study spatial variability of more concrete health outcomes such as injuries, skin conditions and scars with very high degree of certainty; although admittedly, most public health and epidemiological studies have not fully embraced the application of advanced spatial methods probably due to limited understanding of the application of these new spatial methods [47,48,49]. While there is scientific consensus that ecological studies are more reliably conducted over fairly large spatial areas over which multiple socio-economic and environmental factors acting over distinct spatial scales occur in more spatially explicit manner, a few other robust spatial statistical methods which can be applied to study environmental health phenomena over small spatial scales also exist and are widely applied with high degree of success in spatial epidemiology [49,51,53,58,59,60]. Such methods include the one we applied in this study in which a hand-held GPS was used to map out small areas so that scars and other physical/concrete health events were reliably counted on e-waste workers who work within these small spaces on daily basis. Despite the fact that these methods are time consuming, they offer large degree of control and flexibility and are therefore conceivably reliable.

## 5. Conclusions

In conclusion, while the observed injuries were purely random without any role of spatial determinants such as the configuration, slope, topography and other subterranean features of the activity spaces, an association between the injuries and activity type was observed. For this reason, a targeted occupational health and safety (OHS) program will considerably minimize injury rate among the most at-risk group (and in this case; the dismantlers) would help minimize the pervasive injuries among the workers.

## 6. Limitation of Study

This study evaluated the relationship between job-task and injury experience across worker-groups and because some of the workers performed more than one task, it was not possible to objectively make a distinction between scars due to the different job-tasks. However, this limitation was offset by the objective enumeration of scar count which was the main outcome of interest.
